# Difficulties in summing log-normal distributions for abundance and potential solutions

**DOI:** 10.1371/journal.pone.0280351

**Published:** 2023-01-12

**Authors:** Emma J. Talis, Christian Che-Castaldo, Heather J. Lynch

**Affiliations:** 1 Department of Applied Mathematics and Statistics, Stony Brook University, Stony Brook, NY, United States of America; 2 Institute for Advanced Computational Science, Stony Brook University, Stony Brook, NY, United States of America; 3 Department of Ecology and Evolution, Stony Brook University, Stony Brook, NY, United States of America; University of Poonch Rawalakot, PAKISTAN

## Abstract

The log-normal distribution, often used to model animal abundance and its uncertainty, is central to ecological modeling and conservation but its statistical properties are less intuitive than those of the normal distribution. The right skew of the log-normal distribution can be considerable for highly uncertain estimates and the median is often chosen as a point estimate. However, the use of the median can become complicated when summing across populations since the median of the sum of log-normal distributions is not the sum of the constituent medians. Such estimates become sensitive to the spatial or taxonomic scale over which abundance is being summarized and the naive estimate (the median of the distribution representing the sum across populations) can become grossly inflated. Here we review the statistical issues involved and some alternative formulations that might be considered by ecologists interested in modeling abundance. Using a recent estimate of global avian abundance as a case study (Callaghan et al. 2021), we investigate the properties of several alternative methods of summing across species’ abundance, including the sorted summing used in the original study (Callaghan et al. 2021) and the use of shifted log-normal distributions, truncated normal distributions, and rectified normal distributions. The appropriate method of summing across distributions was intimately tied to the use of the mean or median as the measure of central tendency used as the point estimate. Use of the shifted log-normal distribution, however, generated scale-consistent estimates for global abundance across a spectrum of contexts. Our paper highlights how seemingly inconsequential decisions regarding the estimation of abundance yield radically different estimates of global abundance and its uncertainty, with conservation consequences that are underappreciated and require careful consideration.

## Introduction

Log-normal distributions arise frequently in ecology and conservation, often as a means of modeling animal abundance and its attendant uncertainty, but their long right tails can make them difficult to use correctly. If the abundance of animal species is modeled by a log-normal distribution then the aggregate abundance across multiple populations (which could represent different populations of the same species or populations of different species) follows a distribution reflected by the sum of log-normal distributions. Unfortunately, unlike the normal or Poisson distributions, the log-normal distribution is not closed under addition; that is, the sum of log-normal distributions is not itself log-normally distributed. In fact, the sum of log-normal distributions does not follow a known probability distribution at all, and finding a closed form solution for the probability density function for the distribution of sums of log-normals is still an active area of mathematical research [1–4]. Even more seriously, the median of the sum of log-normal distributions is not the sum of the medians of the constituent distributions. Because the median is the measure of central tendency commonly used as a point estimate for skewed distributions, ecologists lack simple or well-known solutions for modeling animal abundance across multiple populations.

### Use of the log-normal distribution in ecology and the modeling of animal abundance

Here we focus on the use of log-normal distributions in the modeling of animal abundance, using as a framework the classic description provided by Clark and Bjørnstad [5] and subsequently developed by other authors [6–8]. The log-normal distribution has several advantages for modeling abundances, including its restriction to non-negative values and—unlike other strictly non-negative distributions such as the Poisson—the ability to tune the variance parameter separately from the mean, and thus to accommodate both under- and over-dispersion with respect to the mean. Thus the continuous log-normal distribution is often preferred to other distributions, including discrete distributions, to model the discrete variable of animal abundance. Two common scenarios leading to the use of log-normal distributions for abundance are models including normally-distributed random variation on growth rate (“process noise,” which represents actual variability of the phenomenon of abundance) and models incorporating log-normally distributed observation error (representing the uncertainty in abundance estimates on account of the method in which estimates are determined). While these two sources of random variation are frequently included together in models of population dynamics [5, 9, 10, pp. 73-76 and 242-244], either one in isolation is sufficient to yield a distribution for abundance that is log-normal. It is important to note, however, that our investigation of this issue is agnostic to the method by which a statistical distribution modeling abundance has been derived.

There are many contexts in which ecologists might want to sum populations across multiple spatial or taxonomic units for the purposes of estimating a point estimate of abundance at larger scales. Most directly, a species may breed in spatially distinct populations, as is common with seabirds, and an estimate of abundance or population trend may require summing these populations [11, 12]. Both metapopulation ecology [13, 14] and landscape demography [15] are concerned with the aggregated dynamics and persistence of spatially distinct populations. In conservation planning, it is a long-standing debate whether it is better to have a single large or several small reserves (the so called SLOSS debate, see [16, 17]), the comparison of which requires some way of summing populations across units. Moreover, ecologists are increasingly looking towards a portfolio approach to conservation, with due consideration for abundance and extinction risk across an ensemble of populations (e.g. [18]). Careful accounting of global abundance could also play an important role for assessment of a species for the International Union for Conservation of Nature Red List, for the designation of Important Bird Areas, or for protection under the US Endangered Species Act. It is important to note that here we focus on applications in which the total across two units should unambiguously equal the sum of the two units taken separately. Notably, the spatial scaling of species richness is not such a case, because species may be common to several units and the total richness (gamma diversity) is not simply the mathematical sum of the richness within each unit (alpha diversity). While analogous concerns may arise in this case as well, here we restrict our attention to the simplest scenario of summing quantities (such as the number of individual organisms) that are unique to each unit.

In its most general form, we will frame our analysis as follows: Consider a collection of different populations, each with abundance modeled by a log-normal distribution, representing the strictly positive number of animals (*X* > 0) within some spatial domain in which the animal is present. Here, we use the word ‘population’ to represent the smallest collection of individuals for which an abundance is estimated. While our analysis applies easily to other scenarios, in the context of our motivating case study [19] each population represents a different species and the aim is to estimate total abundance across all the species. For simplicity, we will refer to this as the ‘global abundance’. We use the standard parameterization of the log-normal distribution *X* ∼ Lognormal(*μ*, *σ*^2^), where *μ* and *σ* represent the mean and standard deviation of the natural logarithm of the random variable (i.e, the abundance of one species), log_*e*_(*X*). On the linear scale, the expected value (or mean) of abundance is given by $e^{\mu +\frac{\sigma^{2}}{2}}$, its median by *e*^*μ*^, and its standard deviation by $\sqrt{\left(e^{\sigma^{2}}-1\right)e^{2\mu +\sigma^{2}}}$.

Estimates for abundance at larger scales should be consistent with the sum of estimates at smaller scales. In the absence of such scale-consistency, our inference becomes sensitive to the scale over which the assessment is made. This leads to a global abundance estimate that no longer corresponds to the individual abundances of each species, or estimates over a larger spatial scale that no longer corresponds to abundances within its constituent components.

### Summing log-normal distributions

The global abundance across multiple species might be estimated in one of two ways, depending on whether the distributions are summarized or summed first. Here and throughout, we use ‘summarize’ to describe the process of obtaining a point estimate of abundance from a distribution while ‘summing’ abundance indicates we are mathematically adding together abundance estimates (in this case, across multiple species) to obtain an estimate of global abundance. As we will see below, the estimate of global abundance obtained by summing abundances may be an entire distribution, incorporating the attendant uncertainty in global abundance, or simply a point estimate.

First, one could summarize each species’ abundance distribution to yield a species-specific point estimate to be added to the other point estimates. While unavoidable in contexts where only point estimates are available for each species, this approach precludes the propagation of uncertainty inherent to each individual species’ estimates to the uncertainty appropriate to the aggregate. Alternatively, one could sum samples from the individual abundance distributions and then summarize the resulting distribution to derive a point estimate for global abundance. In this way, the uncertainty of each individual species’ abundance estimate propagates to the uncertainty in global abundance and a point estimate for global abundance can be easily obtained.

**Fig 1:**
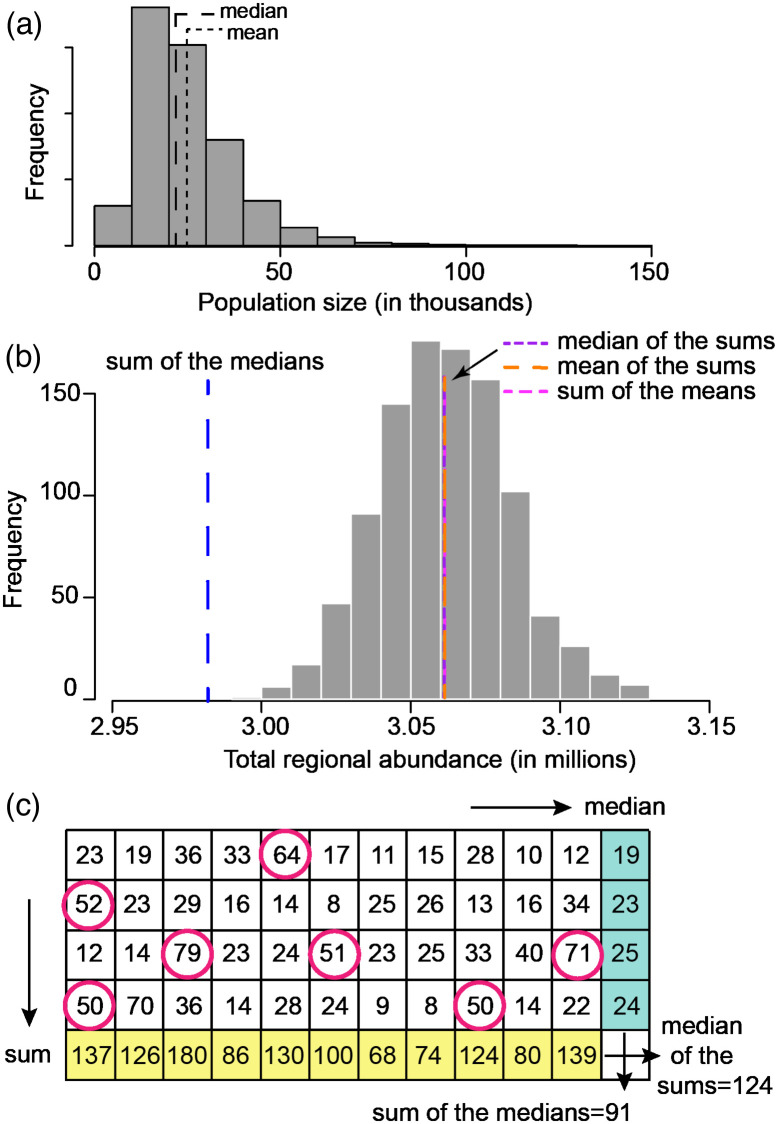
Difficulties in summing log-normal distributions. (a) The mean of the log-normal distribution is pulled right by the long right tail as compared to the median. (b) The distribution of sums of log-normally distributed abundance samples across 1,000 populations each distributed according to (a). The median of this distribution of sums is much larger than the sum of the medians of each individual population, since samples from log-normal distributions will include extreme values from the right-hand tails (circled pink in (c)). (c) This situation is illustrated with 4 populations and 11 samples from the distribution for each population.

At first glance, this second method offers a deceptively straightforward solution to error propagation, involving nothing more than the addition of several distributions for the purposes of arriving at a distribution representing their sum. (As we illustrate in the next section, however, this approach is neither straightforward nor a solution.) While some would argue that the final distribution for abundance is sufficient and, in fact, superior to any measure of central tendency of that distribution, there are many contexts in which a point estimate for abundance is required. In such cases, there are several measures of central tendency that might be used for this purpose. Unlike the normal distribution, which is symmetric, the log-normal distribution is right-skewed, with a long right tail that pulls the mean to the right of the median. Because the mean, median, and mode are identical for the normal distribution, in practice the choice of the most appropriate statistic of central tendency is often irrelevant. For skewed distributions such as the log-normal, however, these three measures can be sharply divergent (Fig 1a).

While rarely discussed explicitly, implicit in the structure of many population models using the log-normal distribution is the assumption that *e*^*μ*^ represents the best estimate of true abundance [20–22]. There are several modeling techniques that allow for the distribution used for modeling abundance to be summarized by the point estimate *e*^*μ*^. One method is to simply model abundance as a log-normally distributed variable and use the median (*e*^*μ*^) as the point estimate (e.g. [23]). As it is often log_*e*_(abundance) that is the response variable being modeled, it is common to back-transform the mean of the normal distribution for log_*e*_(abundance), *μ*, to serve as a point estimate of abundance on the original (linear) scale as *e*^*μ*^ (e.g. [7]). Another approach, described by Hilborn and Mangel [10, pp. 73–76 and 242–244] and popular in the fisheries literature [24–29] involves taking the mean of the shifted log-normal distribution, Lognormal(*μ* − *σ*^2^/2, *σ*^2^), exploiting the fact that the expected value of this distribution is $e^{\mu -\sigma^{2}/2+\sigma^{2}/2}=e^{\mu }$. While these approaches yield equivalent point estimates for the true abundance for a single population, the situation becomes more complex when summing abundance distributions together.

### The median of the sums is not the sum of the medians

As described above, a popular approach for summing multiple log-normal distributions is to simply sum samples from the individual distribution (i.e., each individual log-normal distribution modeling one species’ abundance). An extension of the median as the measure of central tendency for each individual species uses the median of this derived quantity as the best estimate of global abundance. However, the entirety of the distribution for global abundance obtained by summing the samples from the individual species’ distributions is larger than the sum of the medians of the constituent log-normal distributions (Fig 1b). Since the probability of the sum including an anomalously large value from the tail of one of the log-normal distributions (Fig 1c) grows as the number of species being summed increases, so does the difference between these two methods of estimating global abundance. As a result, summing abundances as a derived quantity for each draw from the distribution leads to an estimate larger than would have been obtained by summarizing the individual distributions for each population before summing. This distribution, derived from the sum across species, is not log-normally distributed (in fact, it has no well-described parametric form) and, being centered around the sum of the means of the constituent log-normal distributions, is uniformly too large to represent a reasonable global species sum.

Thus a simple sum of samples drawn from the constituent distributions yields a resulting distribution for global abundance that is too large, with no standard measures of central tendency reflecting the sum of point estimates for the individual populations involved. For these reasons, we argue that the distribution for global abundance obtained by summing draws from the individual abundance distributions is as a whole unreasonable, having been pulled right by the long right tail of the constituent log-normal distributions. Nonetheless, practitioners summing across samples from the individual species-level distributions may not realize that the resulting quantity takes a distribution with unknown properties and moments that no longer reflect the true global abundance. We focus here on illustrating the difficulties inherent in summing log-normal distributions first using a pared down simulation study from which we can explore the conditions under which these challenges are most acute and, secondly, using a more concrete motivating example drawn from a recently published estimate of global avian abundance.

## Simulation study: The extent of the problem

### Simulation study 1: Summing independent and identical log-normal distributions

**Fig 2:**
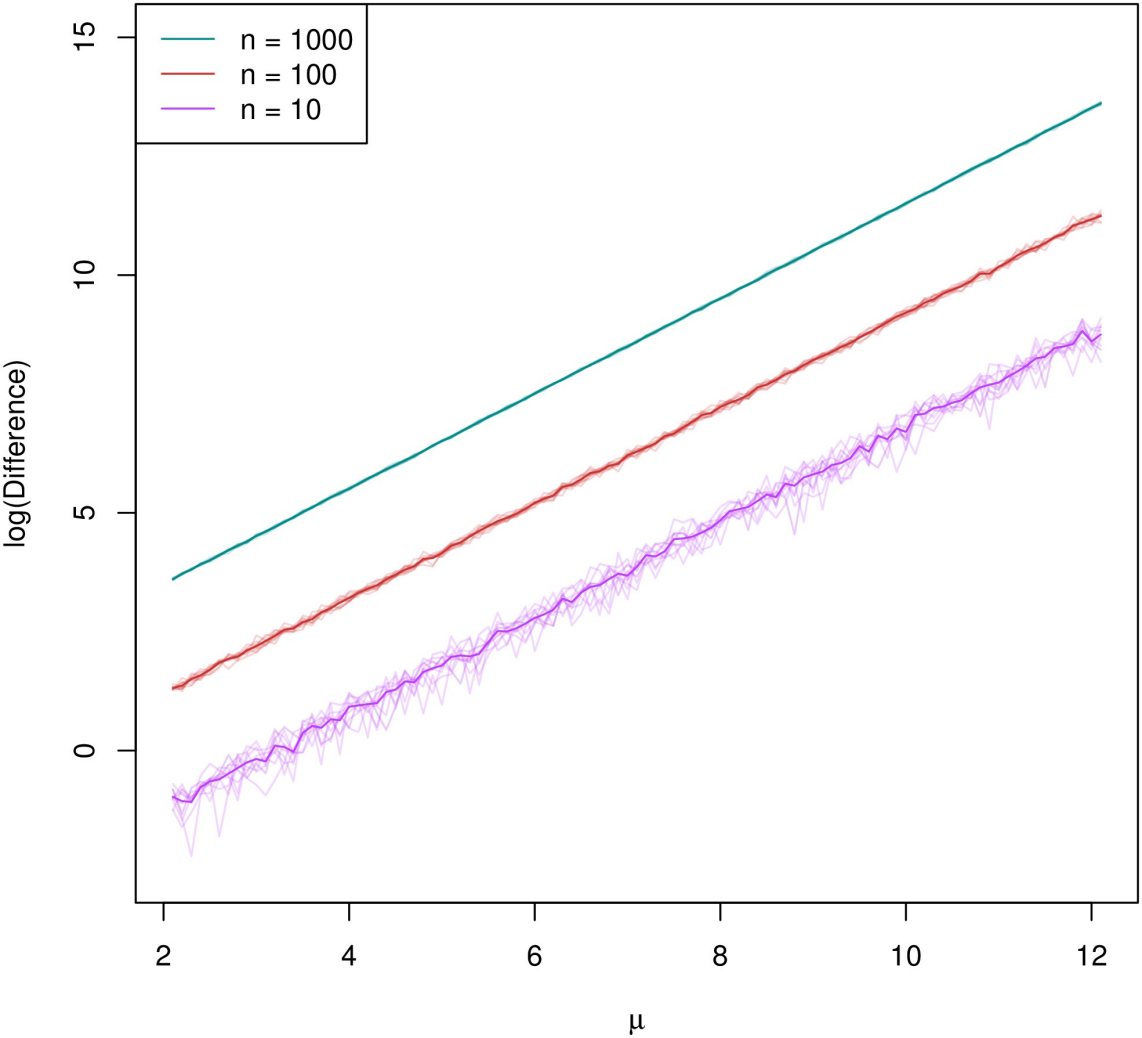
Logged difference between global abundance estimates in simulation study 1. The logged difference in global abundance estimates (i.e., between the sum of the medians of the individual population abundances and the median of the distribution of summed of log-normal populations) for 10, 100, and 1,000 populations modeled using identical and independent log-normal distributions is plotted against the logged-median abundance *μ*. Solid lines represent the mean of the set of 10 iterations for each value of *n*.

To demonstrate the complications inherent to summing log-normal distributions, we first considered a collection of *n* = {10, 100, 1000} populations of animals. Each of these populations’ abundance was modeled as log_*e*_(*N*) ∼ *N*(*μ*, *σ*^2^), where *μ* is the logged-median abundance and *σ* = 0.20 is the standard deviation on the log_*e*_ scale. Thus the *n* log-normal distributions each modeling an individual population’s abundance are identical and independent with these parameters. We performed a suite of such simulations, with each simulation using a different value of *μ* for all *n* log-normal distributions. The range of *μ* values used, 2 ≤ *μ* ≤ 12, represents both small (*e*^2^ ≈ 7 individuals) and large (*e*^12^ ≈ 163, 000 individuals) populations. Setting *σ* = 0.20 yielded log-normal distributions each with an uncertainty of roughly −35% to + 50% (taking the geometric mean, this is roughly ±40%). Each individual abundance distribution consisted of 1, 000 random draws (i.e., realizations from the log-normal distribution). Following the reasoning above, we adopted the sum of medians of each population as the most direct reflection of global abundance and calculated the difference between this estimate of global abundance and that obtained by summing samples across populations to obtain a distribution of sums which is then summarized using the median. For each value of *n*, this difference was calculated 10 times for each value of *μ* and the mean difference was calculated over all 10 iterations. We found that the natural logarithm of this difference between the median of the distribution of sums for global abundance and the sum of the medians of individual populations grew linearly with increasing logged-median abundance *μ* (Fig 2; see S1 Text for mathematical derivation of linearity) and was larger for increasing numbers of populations. This difference grew sharply with increasing *σ* and was greater than 10% with highly uncertain abundances (not shown).

### Simulation study 2: Summing correlated identical log-normal distributions

**Fig 3:**
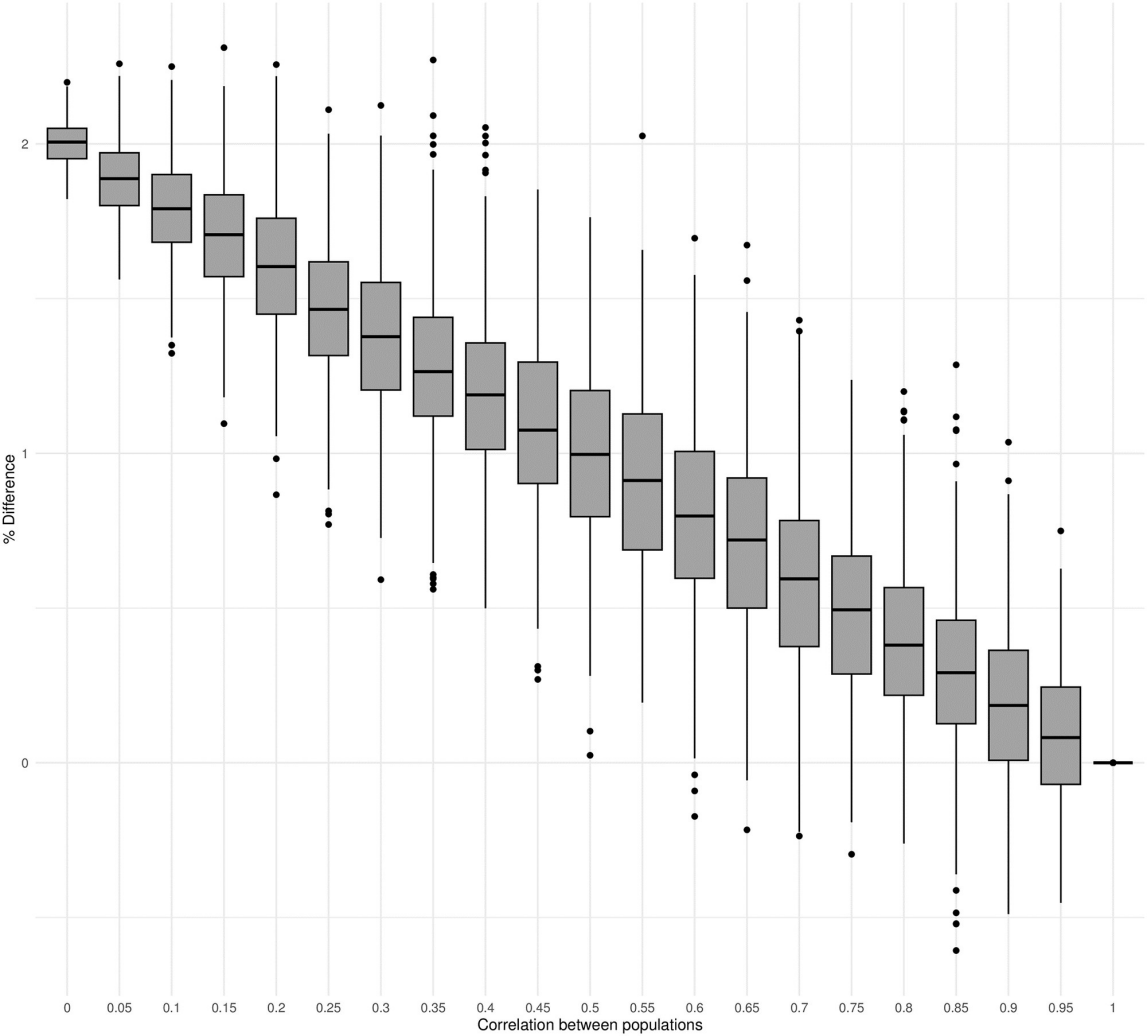
Percent difference between global abundance estimates in simulation study 2. Box plots showing the percent difference in global abundance estimates from 100 simulations of 100 related populations modeled using multivariate identical log-normal distributions with varying correlation coefficient.

We next considered the case were individual populations were modeled using log-normal distributions that were identical but not independent. As above, we calculated the difference between the global abundance estimates. 100 related populations were modeled using identical multivariate log-normal distributions with *μ* = 6 and *σ* = 0.2 and varying correlation coefficient (0 − 1). This simulation was repeated for 100 iterations. As may be expected, the difference between the median of the distribution for global abundance (i.e., the distribution of sums) and the sum of the medians of individual populations shrunk as the correlation among populations increased (Fig 3). There was no difference between these two metrics when the populations were modeled by perfectly correlated log-normal distributions; in this extreme situation, the population collapsed to a single population with abundance modeled by a single log-normal distribution.

### Simulation study 3: Summing independent and non-identical log-normal distributions

**Fig 4:**
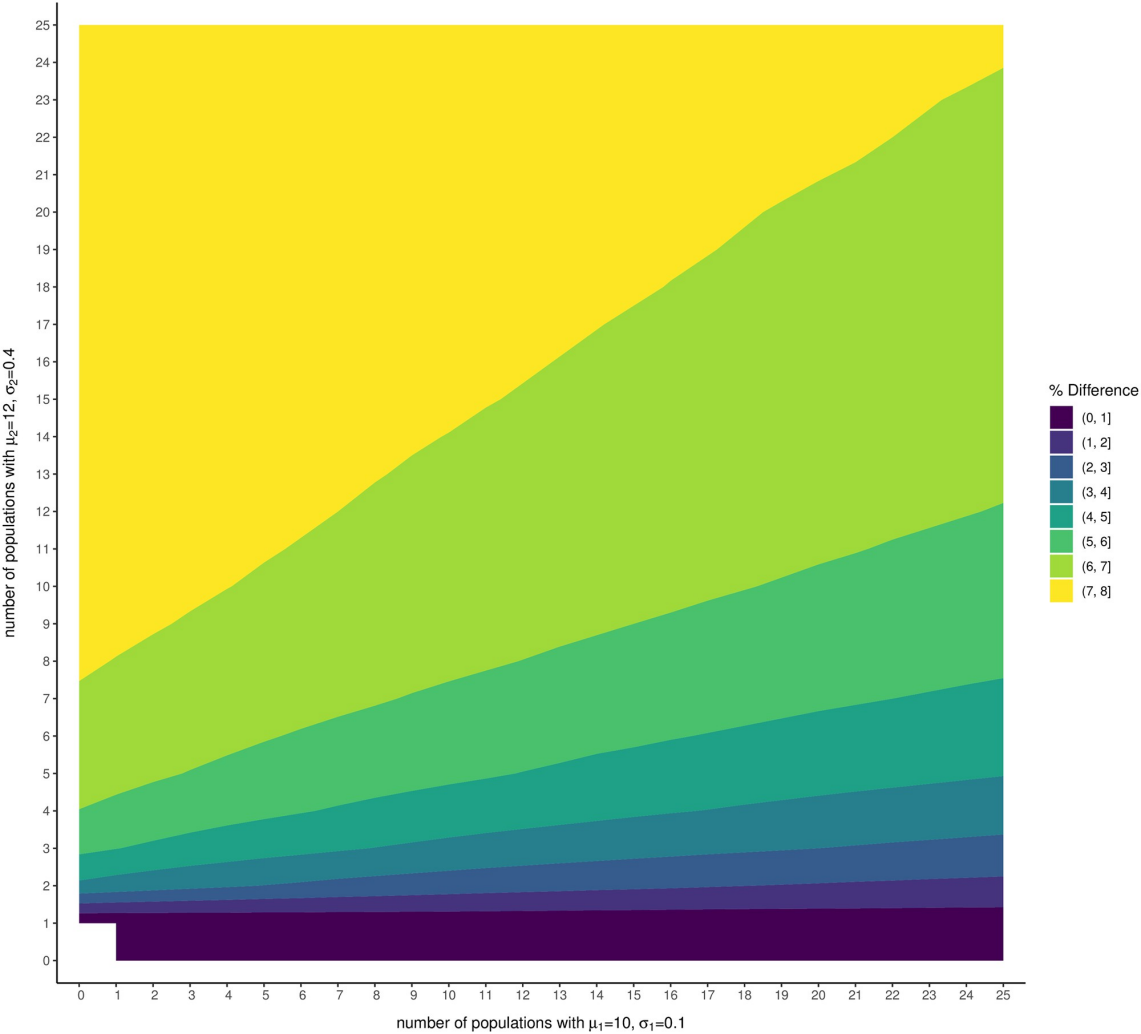
Percent difference between global abundance estimates in simulation study 3. The average percent difference in global abundance estimates is shown for sums of *n*_1_ identical and independent moderate-mean/low-variance log-normal distributions and *n*_2_ populations modeled by identical and independent high-mean/high-variance log-normal distributions.

Lastly, we considered the case of summing non-identical log-normal distributions to understand the extent of the difference between the sum of the medians of the constituent distributions and the median of the distribution of sums when the individual log-normal distributions differ in mean and uncertainty. Specifically, we examined the case in which medium-sized populations known with reasonably high precision (*μ*_1_ = 10, median abundance $e^{\mu_{1}}\approx 22,000$, *σ*_1_ = 0.1) are combined with large populations estimated with low precision (*μ*_2_ = 12, median abundance $e^{\mu_{2}}\approx 160,000$, *σ*_2_ = 0.4). We calculated the difference between the median of the global abundance distribution and the sum of the medians of each population’s abundance distribution for different numbers (*n*_1_ = 1, …, 25 and *n*_2_ = 1, …, 25) of these different sized populations. We found that the percent difference in global population abundance estimates was more severe if the populations being summed were modeled using log-normal distributions that were not identical than compared to the case where the distributions were identical and independent (Fig 4). Further, the inclusion of more populations with high uncertainty caused the largest percent difference.

## Global bird abundance study: A motivating example

We next used the dataset presented in the analysis of global bird abundance by Callaghan et al. [19] to illustrate these same ideas using a real dataset with significant conservation potential [30–32]. In the original study, the abundance of 9,700 individual bird species, each represented by a probability distribution, were summed to generate a distribution (and ultimately, a point estimate) representing global avian abundance [19]. In the context of this principal motivating example, we identified alternative models for species-level abundances yielding a distribution for global abundance closer to the value of the sum of the medians rather than the median of the sums. In this way, we obtained a scale-consistent estimate for global abundance, one that more closely aligned with the sum of the abundance estimates for each individual species (i.e., the sum of medians or, equivalently, $\sum_{i}e^{\mu_{i}}$) and captured the nature of the uncertainty for each species as well. It is worth noting that our use of the Callaghan et al. [19] data is not intended as a criticism of the original paper per se but simply offers an opportunity to illustrate a much more general issue in conservation biology using a dataset that is both recent and conservation relevant.

### Reconstruction of Callaghan et al. data

In Callaghan et al. [19], the authors provide abundance estimates for each bird species summarized by the median and lower and upper bounds of the 95% CIs on log_10_(Abundance), which are assumed to be normally distributed (see Methods and Dataset S1 of [19]). We used the standard deviation for these normal distributions ((log_10_(lower limit) + log_10_(upper limit))/2)/1.96) and drew 10,000 random realizations from a normal distribution with mean equal to log_10_(median) and the calculated standard deviation. We then exponentiated (base 10) these realizations to yield a sample from the abundance distribution on the linear scale and fit a log-normal distribution (base *e*) to that sample, obtaining values for the parameters *μ* (the logged-median abundance, or mean on the log_*e*_ scale) and *σ* (the standard deviation of the log_*e*_ scale) of each log-normal distribution. We then obtained 10,000 random draws from each of these log-normal distributions, each used to model the abundance of a given bird species. This procedure allowed us to move from the original authors’ [19] choice of a base-10 logarithm to a more traditional base-*e* logarithm and to generate a dataset that mirrors the original study’s underlying population distributions [19].

Though our use of this dataset is intended for illustration purposes only, we wanted to confirm our reconstruction of the data used in Callaghan et al. [19], as doing so allows us to make direct comparisons between the estimates presented in the original study [19] and those resulting from the alternative summing and summarizing approaches addressed below. As such, we recreated each of the plots in Callaghan et al.’s [19] Fig 2A and 2B (see S2 and S3 Figs, respectively); we captured the data well, with a similar global species abundance distribution and individual distributions for species abundance. The average global population estimate over all 9,700 species was 5.2 million and the median estimate was 450,000, each equal to the estimate reported by Callaghan et al. [19].

We then generated a distribution for global abundance of birds by simply summing the distributions for individual abundance. To illustrate why choices regarding the distribution, summing approach, and measure of central tendency used all have consequences for point estimates of global abundance, we then calculated this sum using a suite of different approaches that might be used by ecologists looking for scale-consistent abundance estimates.

### Alternative solutions

In their analysis, Callaghan et al. [19] circumvent the issue of the median of the sum of log-normal distributions being unequal to the sum of the constituent medians by sorting the samples within the distributions for each bird species before summing them. In other words, the smallest value in their distribution for global bird abundance represents the sum of the smallest values in the distribution for each bird species. As described by Callaghan et al. [19], this sorted summing “ensure[s] that the likelihood of values particular to each species correspond[s] with one another, therefore ensuring that the middle values correspond to those with the highest likelihood”. Rephrased in the context of our analysis, this sorted summing ensures that the median of the resulting summed distribution is the sum of the medians. Using the log-normal distributions generated above, we followed this sorted summing procedure to obtain a second distribution for global abundance.

Another approach to ensure a scale-consistent distribution for global abundance is found in the aforementioned method described by Hilborn and Mangel [10, pp. 73–76 and 242–244], which shifts the log-normal abundance distribution for each species such that the mean—which has the desired additive property but is too large to use as the point estimate for abundance—replaces what was previously the median of the distribution. This model produces scale-consistent estimates for individual species’ abundances (for which the point estimate is now the mean) and the total distribution for global bird abundance (the mean of which is equal to the sum of the means of each individual species’ abundance). While this adjustment has been used previously [11, 33, 34] and is, in fact, quite standard within the fisheries literature [24–29], there has been little discussion of its use in the context of population biology despite its utility in this context [11, 12]. We used this approach to obtain a third distribution for global abundance, using the parameters of the log-normal distributions calculated above with the Callaghan et al. data [19] to generate shifted log-normal distributions as $N∼\text{Lognormal}(\mu -\frac{\sigma^{2}}{2},\;\sigma^{2})$, and then summed them sample-wise. Note that, for this procedure and those that follow, we sum constituent distributions simply without sorting them first.

The constituent log-normal distributions for individual species abundance may also be replaced by the (0, ∞) truncated normal distribution with the same mean and standard deviation (on the linear scale). Note that even with no change in *μ* or *σ*, the expectation of this zero-truncated distribution is greater than *μ* [35]. Accordingly, we obtained a fourth distribution for global abundance by summing zero-truncated normal distributions for each species as $N∼\text{trunc}N(e^{\mu },\;\left(e^{\sigma^{2}}-1\right)e^{2\mu +\sigma^{2}},\;0,\;\infty )$, where *μ* and *σ* are the parameters (on the log_*e*_ scale) of the uncorrected log-normal distribution calculated above and $\left(e^{\sigma^{2}}-1\right)e^{2\mu +\sigma^{2}}$ is the variance of that log-normal distribution on the linear scale.

Another correction involves replacing the log-normal distributions for abundance with a rectified normal distribution with mean *e*^*μ*^ and standard deviation *σ*. As opposed to the zero-truncated normal distribution, the rectified normal moves the statistical support for negative values to zero, leaving the median in most cases unchanged [36]. This rectification can take place at the level of individual populations (i.e., before summing) or can be done after summing. We present both methods below. These methods produce more scale-consistent distributions for global abundance by ensuring the median of the resulting distribution of sums is equal to the sum of the point estimates of the individual log-normal distributions representing single species’ abundances. We generated our final two distributions for global abundance using normal distributions for each species as $N∼N(e^{\mu },\;\left(e^{\sigma^{2}}-1\right)e^{2\mu +\sigma^{2}})$, where *μ* and *σ* are the parameters of the uncorrected log-normal distribution calculated above. For the rectify-then-sum procedure, we rectified the individual distributions by taking the maximum max(0, *x*) for each random draw *x* from the normal distribution and then summed the distributions sample-wise to obtain the distribution for global abundance. For the sum-then-rectify procedure, we sum the unrectified normal distributions sample-wise and then took the maximum max(0, *s*) for each sum *s* in this global abundance distribution.

### Performance of alternative solutions

**Fig 5:**
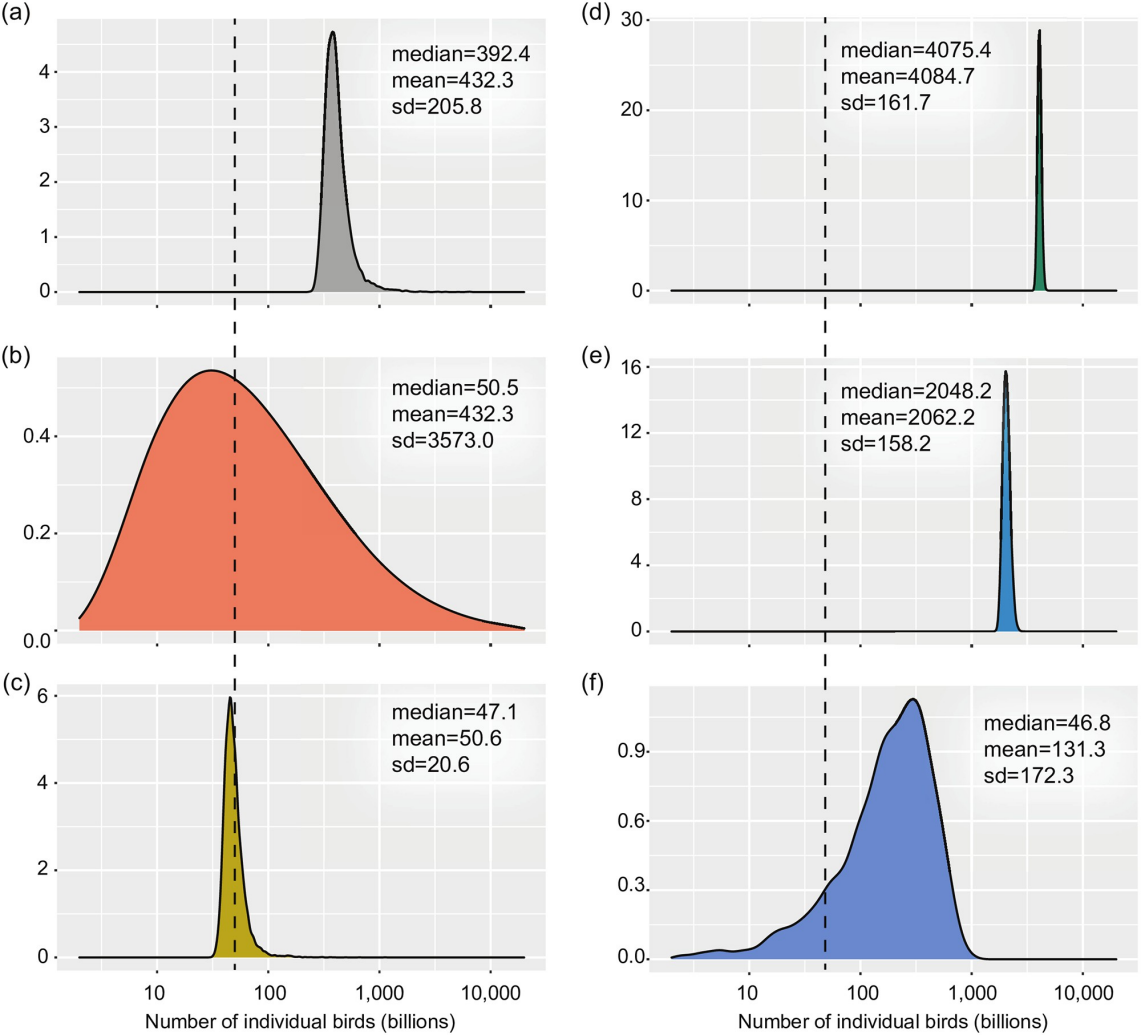
Performance of alternative solutions on global bird abundance data. The total distribution of the number of individual birds in the world, calculated by summing all species-specific abundance distributions for 9,700 bird species using various corrections. The median, mean, and standard deviation (in billions) are shown for each distribution. The dotted vertical line indicates the sum of individual species abundance estimates (50 billion). (a) uncorrected sum of log-normals; (b) sort-then-sum of log-normals; (c) shift-then-sum of log-normals; (d) truncate-then-sum of normals; (e) rectify-then-sum of normals; (f) sum-then-rectify of normals. Details in the text.

As described above, in our re-analysis of the Callaghan et al. bird abundance data [19], we compared the summing of uncorrected log-normal distributions for individual species’ abundance against five alternative approaches (Fig 5; note the log_10_ scale). Central tendencies used for the global abundance distributions are the following: median for sort-then-sum log-normals; mean for the shift-then-sum log-normals; mean for the truncate-then-sum normals; median for the rectify-then-sum normals; and median for the sum-then-rectify normals. Note that, in Fig 5, histogram bin widths underlying the kernel density plots shown are of equal width on the log_10_ scale used in this figure; as a result, the bulk of the area under the curve as displayed falls to the right of the distributions median. Plotted on a linear scale, the area under the curve would be equally partitioned to the right and left of the median. Here we have used the log_10_ scale both to match Fig 5b to Callaghan et al.’s original study [19] and because the differences between the methods are difficult to visualize on a linear scale.

The global abundance distribution obtained using the sort-then-sum log-normals method used by Callaghan et al. (shown in Fig 5b) is a direct reproduction of Fig 2C in the original paper [19]. As was the motivation behind our study, the global abundance distribution obtained by simply summing uncorrected log-normal distributions for individual species’ abundance (Fig 5a) was far from the sum of all species’ abundance estimates in its entirety. Three alternative methods of generating a global abundance distribution had measures of central tendency close to the sum of the medians of the individual species’ abundance distributions (cited by Callaghan et al. as 50 billion [19]): sort-then-sum log-normal distributions (Fig 5b), shift-then-sum log-normal distributions (Fig 5c), and sum-then-rectify normal distributions (Fig 5f). Of these global abundance distributions [19], the one obtained using the sort-then-sum method (Fig 5b) had the largest standard deviation, 17 times larger than the standard deviation of the global abundance distribution obtained by summing uncorrected log-normal distributions (Fig 5a) and more than 20 times larger than that of any other corrective method’s resulting distribution. The global abundance distributions generated using truncate-then-sum (Fig 5d) and rectify-then-sum normal distributions (Fig 5e) were entirely larger than the sum of the medians of the individual populations, a problem even more extreme than for the global abundance distribution obtained by merely summing uncorrected log-normal distributions (Fig 5a).

#### Performance of alternative solutions in simulation study

**Fig 6:**
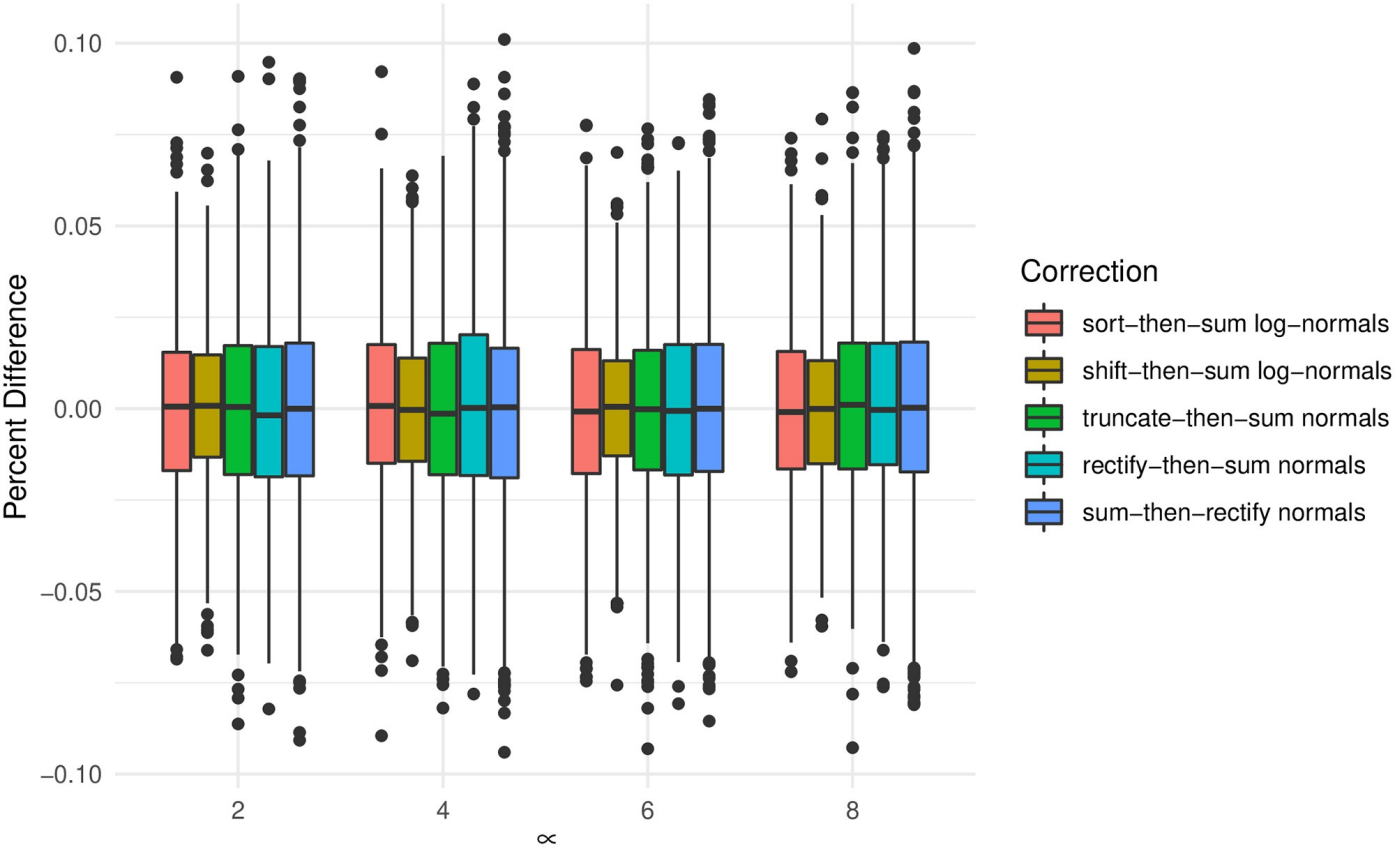
Performance of alternative solutions on simulated data. Box plots for the percent difference between the sum of empirical population medians (*ne*^*μ*^) and the point estimate for global abundance obtained using each of the five corrective models for different values of logged-median abundance *μ* (box plot whiskers go to 1.5 times the interquartile range from the first and third quartiles).

We also calculated the percent difference between the sum of the abundance estimates for each individual species and the central tendency (either mean or median, see above) of the global abundance distribution generated by each of the alternative procedures presented above for the earlier simulation study. For each of the five procedures, 100 fictive populations are simulated using the given probability distribution with *μ* = {2, 4, 6, 8} and *σ* = 0.2 consisting of 10, 000 random draws. The distributions modeling each population’s abundance are assumed to be identical and independent. The global abundance distribution was generated by summing these individual population abundance distributions, and an estimate for global abundance was obtained. This was repeated for a total of 100 ensembles for each procedure. All five alternative approaches introduced above allowed for scale-consistent estimates of global abundance to be obtained in this case (Fig 6). The percent difference between the point estimate for global abundance and the sum of the medians of each individual population was negligible for each corrective procedure (compared to an average percent difference of 2% obtained summing the uncorrected sum of log-normal distributions). Across 100 ensembles, this percent difference was the least variable for the shift-then-sum log-normal procedure.

## Discussion

We have shown here the issues involved in summing log-normal distributions modeling abundance. These issues arise in a variety of contexts, regardless of the method by which a log-normal distribution modeling abundance has been derived. It is worth noting, however, that this distribution is often a posterior distribution estimated through a Bayesian analysis. While our examination of our findings includes some points specific to this Bayesian approach, it is important to remember that the issues of summing and summarizing distributions are generic and apply equally to both Bayesian and frequentist approaches.

### The choice of point estimate

Our discussion of the challenges of summing log-normal distributions centers around the use of the median as the “best” measure of central tendency to summarize a log-normal distribution modeling abundance. It is worth reflecting on the advice provided to ecologists for this choice. The mode is, by definition, the value of the distribution that is most likely. In a Bayesian analysis, the mode is also the value most similar to the maximum likelihood estimate when the prior is uninformative. The mean and median minimize the sum of squared error and the sum of absolute error, respectively. These basic facts, however, are unhelpful for most practitioners in choosing an appropriate point estimate.

Perhaps surprisingly, ecological modeling textbooks provide scant advice on the matter and are largely agnostic on the choice between mean, median, and mode when deciding how to summarize a distribution, presenting them as largely interchangeable choices [37, 38]. Gelman et al. [38] present one example in which the distribution of a parameter includes unphysically negative values (akin to a normal distribution for abundance) and note that “the marginal posterior mean is not always a good summary of inference about a parameter…because the posterior mean includes the cases where [the parameter] is negative.” Lunn et al. [37] touch on this briefly as well, noting that “[t]here have been limited ‘guidelines’ for reporting Bayesian analyses” and that “[w]here possible, full posterior distributions should be given…particularly for skewed distributions.” While we agree that the presentation of the full distribution is important, it is no substitute for providing a concrete point estimate of abundance in conservation contexts, particularly when providing information to policymakers without specialized statistical knowledge.

Finally, as noted above, the uncertainty reflected in a distribution for abundance often arises from observation error. Using the median as the point estimate for abundance implies that under-counts and over-counts are equally likely. Such an assumption might be reasonable for many sampling methodologies. Additionally, the mean ($e^{\mu +\sigma^{2}/2}$) changes as a function of a survey’s precision. Thus, when using the mean as the point estimate of true abundance, a stable population surveyed over time would appear to decline as an artifact of declining measurement error over time (as might happen, for example, with improved survey methods). The use of the median avoids this logical inconsistency whereby different survey processes would change the underlying population abundance. For both of these reasons, the use of the median is often preferred over the mean when observation error is involved.

### Callaghan et al.’s approach

The propagation of uncertainty from several log-normally distributed populations to a distribution representing their sum is a surprisingly difficult task that has garnered far too little attention given the consequences for conservation biology [13, 15, 16, 39]. The approach taken by Callaghan et al. [19] to sort the samples making up each individual species’ log-normal abundance distribution before summing is, to our knowledge, an unusual choice. While this summing procedure did generate scale-consistent estimates for global abundance, it did so at the expense of the resulting distribution’s variance. The standard deviation of the global abundance distribution became grossly exaggerated under the sort-then-sum procedure, with a median of 50 billion, a mean of 431 billion, and a range of 256 trillion (Fig 5b). This degree of uncertainty will render the estimates for global abundance useless in many conservation contexts, as was noted by critics [40].

Earlier concern regarding Callaghan et al.’s [19] methodologies, however, focused on the acutely uncertain and often biased constituent distributions modeling individual species’ abundance [40, 41]. Our analysis identifies an important and previously unrecognized driver of uncertainty in their final distribution for global bird abundance (the sort-then-sum procedure), and we have found that alternative approaches result in distributions for global bird abundance with dramatically lower uncertainty. The authors’ choice of the sort-then-sum procedure and the associated uncertainty is not discussed in detail in the original study, nor is the difference between the mean and median as alternative point estimates. While modifying the method by which abundance distributions are summed will not address all of the concerns raised, it is important to separate out sources of uncertainty so that each can be considered thoughtfully and minimized where possible.

### The choice of alternatives: Context matters

In our re-analysis of the global bird abundance data [19], two alternative models (i.e., replacing the log-normal distributions modeling individual species abundance with shifted log-normal distributions or with normal distributions that are rectified after summing) generated distributions for global abundance that were consistent with the sum of the estimates from the individual distributions for species abundance. Though they produced similar point estimates (using the mean and median, respectively), these two methods generated different distributions for global abundance (Fig 5c and 5f). The distribution obtained using the shift-then-sum log-normal distributions procedure had the smallest variance of any global abundance distribution, with a standard deviation about 10 times smaller than that of any other. The distribution obtained using the sum-then-rectify normal distributions procedure had a median close to the sum of the individual species’ abundance estimates but the mean of the distribution is pushed right after the distribution is rectified following summing. Additionally, the global abundance distribution generated by summing zero-truncated normal distributions for individual species’ abundance (Fig 5d) had a mean (and median) much larger than even the distribution obtained using raw log-normal distributions on account of the increased expected values of the constituent truncated distributions [35]. Likewise, the global abundance distribution obtained using the rectify-then-sum normal distributions procedure (Fig 5e) was entirely too large, since the individual distributions representing species’ abundances were heavily skewed by the rectification process. Clearly, these two procedures led to extreme scale-inconsistency for the Callaghan et al. data [19]. However, these issues may be less severe in a context where fewer, less abundant, or less uncertain individual species are being summed. In the case that species with more modest abundance uncertainties are modeled using identical and independent distributions, our simulation study showed that all alternative methods presented here produced scale-consistent point estimates for global abundance (Fig 6). Thus, while using shifted log-normal distributions worked well in both contexts shown here, the suitability of these alternative procedures should be assessed carefully for specific applications.

While we focus here on the use of log-normal distributions in the modeling of animal abundance, which is an easily conceptualized problem with clear conservation importance [7, 13, 16–20, 42, 43], there are many contexts in which ecologists might be trying to sum unique log-transformed items or measures across multiple temporal, spatial, or taxonomic scales. In addition to animal abundance, examples include quantities like biomass, mortality, and rainfall [44–46]. The use of the sort-then-sum log-normals, shift-then-sum log-normals, truncate-then-sum normals, rectify-then-sum normals, and sum-then-rectify normals procedures provided the scale-consistency that is desired in some scenarios. It is of the utmost importance, however, to note that each of these approaches modified the distribution of the sum in ways that can impact inference if the populations are very small, the uncertainties are very large, or if the analysis requires a careful consideration of distribution statistics other than the mean or median. The choice among these approaches depends on the specific application and whether inference hinges only on a measure of central tendency or whether other statistics, such as specific quantiles, may be needed. While the use of shifted log-normal distributions works well in both extreme examples presented here and has been used previously [11, 12, 33, 34], it should not be adopted without thoughtful consideration of its impact in the context of the specific conservation question being asked.

### Data-deficient species

As we have shown, the use of the log-normal distribution to model abundances caused the most significant challenges for summing when population estimates were highly uncertain, exactly the one might face with highly data-deficient species. Even for species that have been surveyed in detail, the uncertainty surrounding abundances can become very large over an extended period of time without observation data, since, as described by Clark et al. [5], “as the interval between observations widens, the variability contributed by process error increases correspondingly”. In these cases, the most current understanding of abundance for any given population may be highly uncertain, and even small numbers of populations with such imprecise abundance estimates can contaminate global abundances unless care is taken to carefully choose the summing procedure and the measure of central tendency used as the reported point estimate. We also showed the challenge of summing abundances over multiple populations was reduced, but not eliminated, when abundances were correlated across populations. While such correlations may be present to a modest extent in a spatial context, in which neighboring populations may have correlated errors, such correlations would be absent in a context like that presented by Callaghan et al. [19]. In any case, it was only in the most extreme and highly unrealistic situation of populations with perfectly correlated errors that this difference actually collapsed to zero; in such a scenario, the populations are functionally one single population.

## Conclusions

At its heart, our analysis calls into question under what conditions the log-normal distribution is appropriate for modeling animal abundance, particularly in cases where the distribution represents a Bayesian posterior distribution directly interpreted as the degree of belief one has in different values of abundance as true abundance (these more philosophical issues are discussed in S2 Text). Our choices of distributions for modeling animal abundance are extremely limited; our wish list for the ideal distribution includes one that is discrete, non-negative, closed under addition, and has a variance that can be tuned separately from the mean. We are aware of no parametric distribution that satisfies all four of these criteria [47], and thus it is incumbent on ecologists to select distributions whose shortcomings have the least impact on the problem at hand. In fact, many popular distributions are both skewed and not closed under addition, including the negative binomial distribution, and these also require considerable care in their use (see S3 Text). While the log-normal distribution emerges naturally from the exponential growth process and its non-negativity is convenient, its right skew can be severe when uncertainty is large and its use for conservation relevant analyses should be undertaken with care and careful consideration. The accurate estimation of abundance is important in its own right and we hope that our analysis will encourage ecologists to think carefully about how their choices in modeling abundance across different spatial or taxonomic scales may impact the final estimates provided to stakeholders.

## Supporting information

S1 Fig Parameter values used in the simulation study and global bird abundance study. Log-normal parameter values *μ* and *σ* used in both the simulation study and the re-analysis of Callaghan et al.’s global bird abundance data.
(PDF)

S2 Fig Reconstructed global species abundance distribution for global bird abundance. The global species abundance distribution, calculated using the median of each species’ simulated abundance distribution (each a log-normal distribution) for the re-analysis of the global bird abundance data. A constant 1 is added for species predicted to have zero abundance. Reproduction of Fig 2A in Callaghan et al. (shown on the same scale, log_10_).
(PDF)

S3 Fig Examples of reconstructed species’ simulated abundance distributions for global bird abundance study. Species shown, from top to bottom: Ring-billed Gull; Green Heron; Northern Wheatear; Ashy Prinia; Osprey; Acorn Woodpecker; Yellow-tailed Black-Cockatoo; and Midget Flowerpecker. Reproduction of Fig 2B in Callaghan et al. (shown on the same scale, log_10_, in millions).
(PDF)

S1 Text Derivation of linearity of difference in global abundance estimates. (PDF)

S2 Text How Bayesians interpret the tail of the skewed distributions. (PDF)

S3 Text Summing negative binomial distributions. (PDF)
