# Supplementary figures and images for: Difficulties in summing log-normal distributions for abundance and potential solutions
Source: PLoS One. 2023 Jan 12;18(1):e0280351. doi: 10.1371/journal.pone.0280351 (PMC9836268; doi:10.1371/journal.pone.0280351)

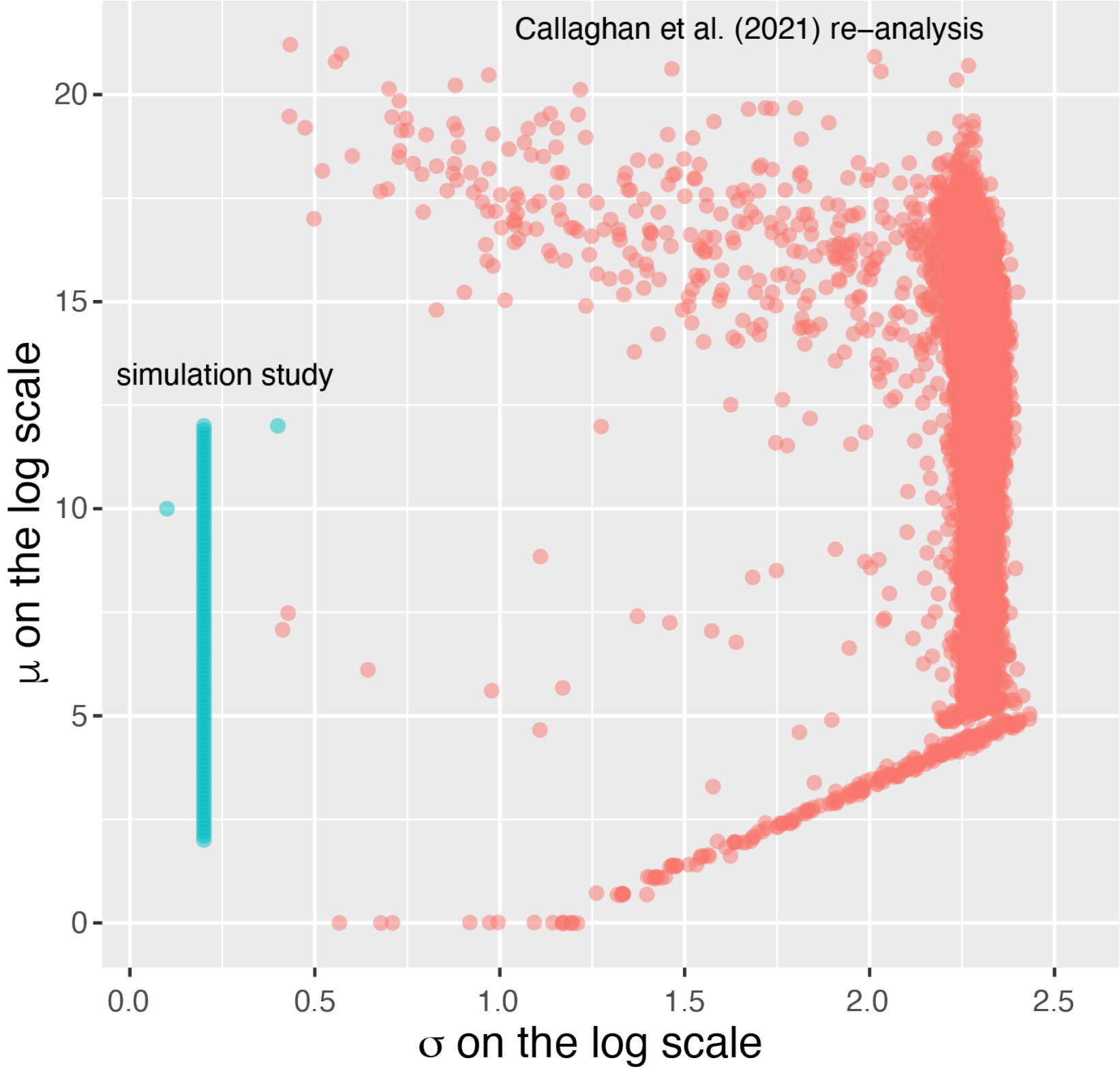

Supplement: S1 Fig — Log-normal parameter values μ and σ used in both the simulation study and the re-analysis of Callaghan et al.’s global bird abundance data. (PDF) [file pone.0280351.s001.pdf]

Species Abundance Distribution

100,000,000

100,000

100

0

200

400

600

800

Number of species

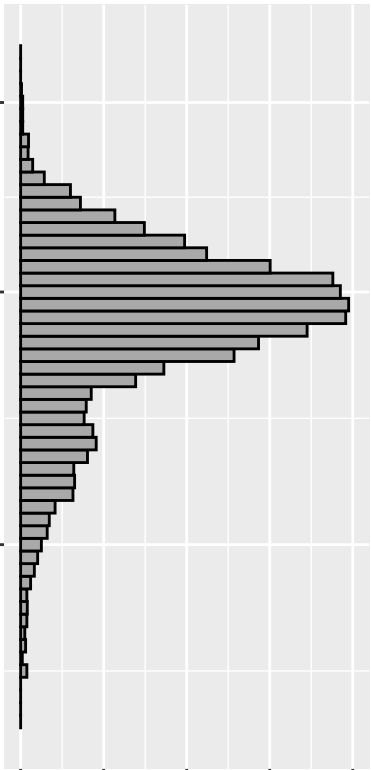

Supplement: S2 Fig — The global species abundance distribution, calculated using the median of each species’ simulated abundance distribution (each a log-normal distribution) for the re-analysis of the global bird abundance data. A constant 1 is added for species predicted to have zero abundance. Reproduction of Fig 2A in Callaghan et al. (shown on the same scale, log10). (PDF) [file pone.0280351.s002.pdf]

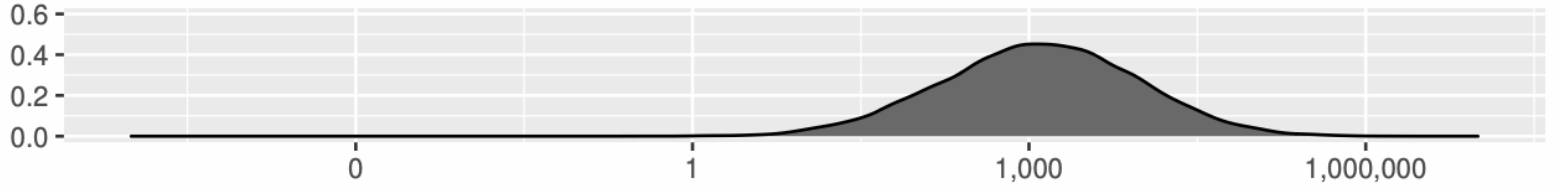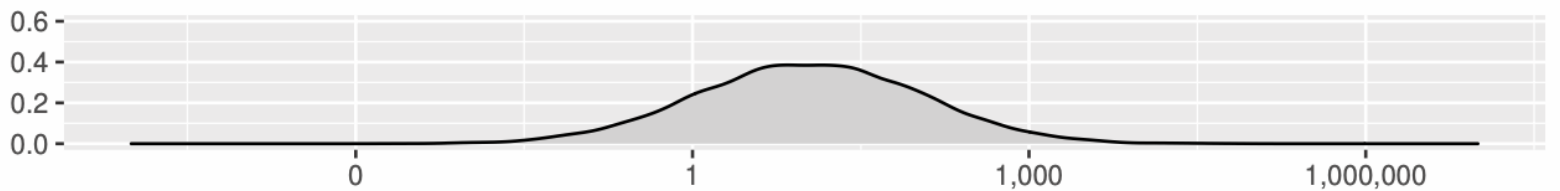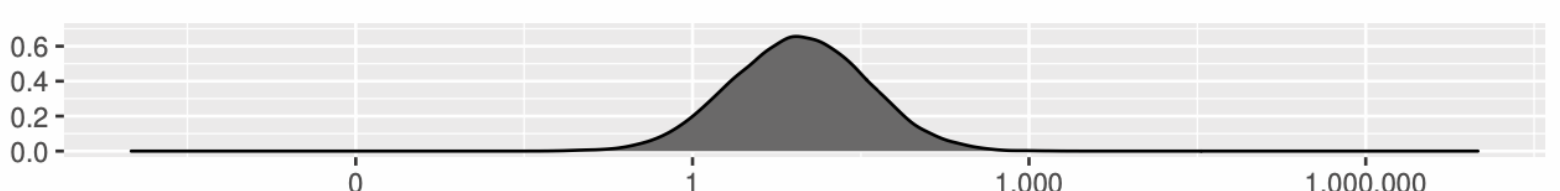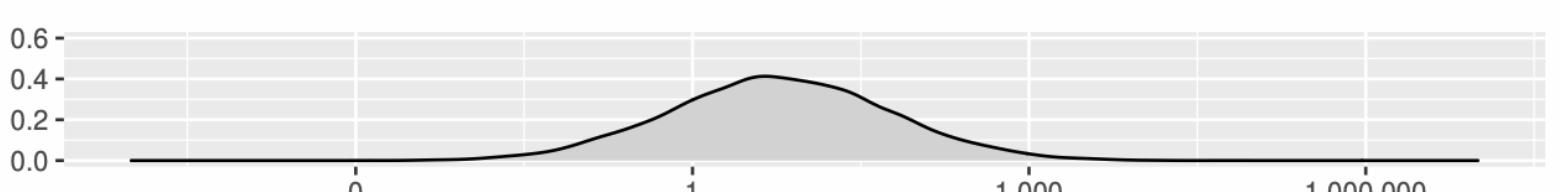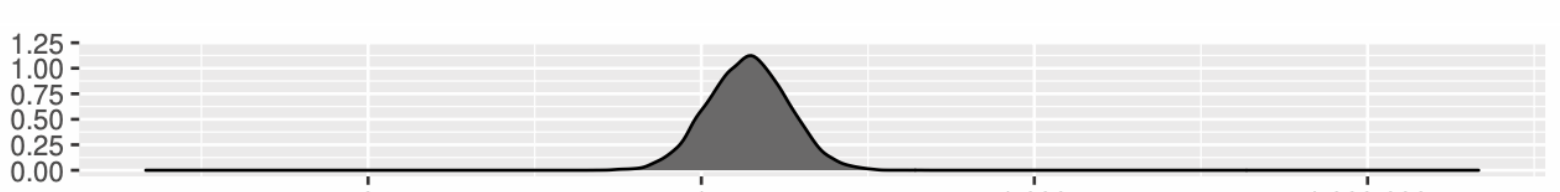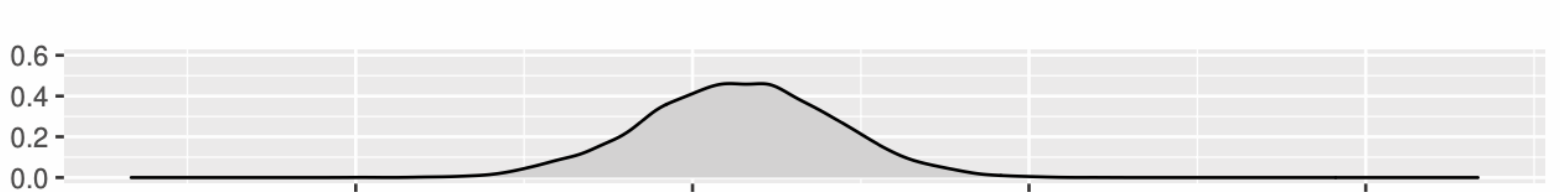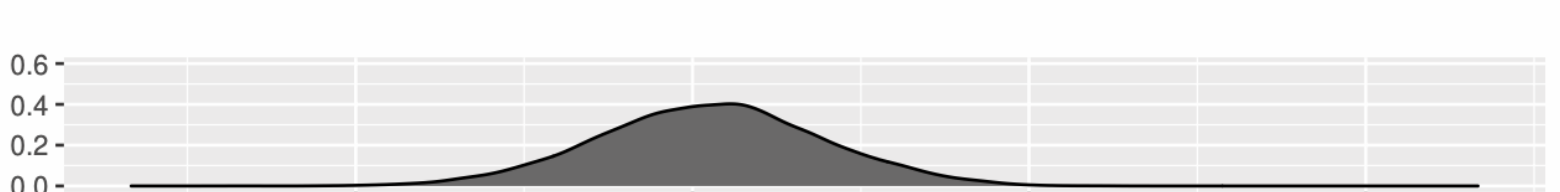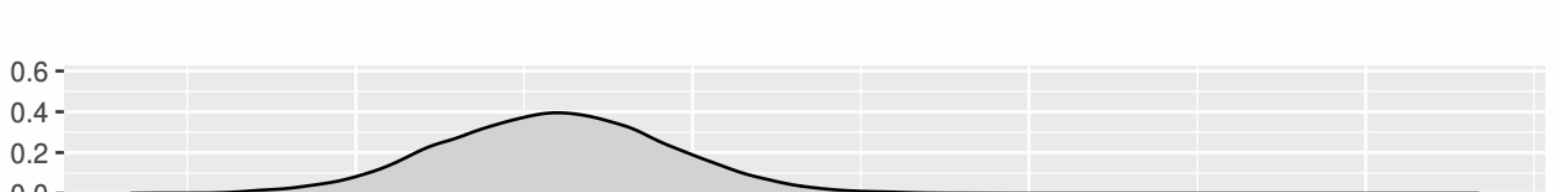

Number of individual birds (millions)

Supplement: S3 Fig — Species shown, from top to bottom: Ring-billed Gull; Green Heron; Northern Wheatear; Ashy Prinia; Osprey; Acorn Woodpecker; Yellow-tailed Black-Cockatoo; and Midget Flowerpecker. Reproduction of Fig 2B in Callaghan et al. (shown on the same scale, log10, in millions). (PDF) [file pone.0280351.s003.pdf]
