# Supplementary material for: Difficulties in summing log-normal distributions for abundance and potential solutions
Source: PLoS One. 2023 Jan 12;18(1):e0280351. doi: 10.1371/journal.pone.0280351 (PMC9836268; doi:10.1371/journal.pone.0280351)
Supplement: S1 Text — (PDF) [file pone.0280351.s004.pdf]

S4 Text for

Difficulties in summing distributions for abundance and potential solutions

Emma Talis[1,2], Christian Che-Castaldo[2], Heather J. Lynch[2,3]

[1]Department of Applied Mathematics and Statistics, Stony Brook University

[2]Institute for Advanced Computational Science, Stony Brook University

[3]Department of Ecology and Evolution, Stony Brook University

Derivation of linearity of difference in global abundance estimates

Consider $n = \{10, 100, 1000\}$ i.i.d. log-normally distributed populations $N_1, N_2, ..., N_n$ each satisfying $\log(N_{i_t}) \sim N(\mu, \sigma^2)$. Let the D be equal to the logged-difference between the median of the posterior for the aggregate sum of populations and the sum of the individual population medians. Then

$$D = \log(M - S), \tag{1}$$

where M is the median of the posterior for $N = N_1 + N_2 + ... + N_n$, the aggregate sum of populations and S is the sum of the individual medians of each population.

The sum of n i.i.d. log-normal random variables N has no closed form probability density function. However, the Fenton-Wilkinson (FW) approximation produces a commonly used estimate for the PDF of N [1]. According to the FW approximation [2], N can be approximated by a log-normal PDF with parameters μ_N and σ_N^2 such that

$$\sigma_N^2 = \frac{\log(e^{\sigma^2 - 1})}{n} + 1, \tag{2}$$

$$\mu_N = \log(ne^{\mu}) + \frac{1}{2}(\sigma^2 - \sigma_N^2). \tag{3}$$

Thus the median of N is given approximated by

$$\begin{aligned} M = \mathrm{med}(N) &\approx \frac{\log(ne^{\mu}) + \frac{1}{2}(\sigma^2 - \sigma_N^2)}{e^{\sigma_N^2/2}} && (4) \\ &= \frac{\log(ne^{\mu}) + \frac{1}{2}(\sigma^2 - (\frac{1}{n}\log(e^{\sigma^2 - 1}) + 1))}{e^{(\frac{1}{n}\log(e^{\sigma^2}-1)+1)/2}} && (5) \\ &= \frac{\log(n) + \log(e^{\mu}) + \frac{1}{2}(\sigma^2 - (\frac{1}{n}\log(e^{\sigma^2 - 1}) + 1))}{e^{(\frac{1}{n}\log(e^{\sigma^2}-1)+1)/2}} && (6) \\ &= \frac{\log(n) + \mu + \frac{1}{2}(\sigma^2 - (\frac{1}{n}\log(e^{\sigma^2 - 1}) + 1))}{e^{(\frac{1}{n}\log(e^{\sigma^2}-1)+1)/2}} && (7) \\ &= \frac{\mu}{e^{(\frac{1}{n}\log(e^{\sigma^2}-1)+1)/2}} + \frac{\log(n) + \frac{1}{2}(\sigma^2 - (\frac{1}{n}\log(e^{\sigma^2 - 1}) + 1))}{e^{(\frac{1}{n}\log(e^{\sigma^2}-1)+1)/2}}. && (8) \end{aligned}$$

Setting $a = \log(n) + \frac{1}{2}(\sigma^2 - (\frac{1}{n}\log(e^{\sigma^2-1}) + 1))$ and $b = e^{(\frac{1}{n}\log(e^{\sigma^2}-1)+1)/2}$, 8 reduces to

$$M \approx \frac{1}{b}\mu + \frac{a}{b}. \qquad (9)$$

Since the median of each log-normally distributed population N_i is equal to e^{μ}, the value of S can be found easily:

$$\begin{aligned} S &= \sum_{i=1}^{n} \mathrm{med}(N_i) \\ &= \sum_{i=1}^{n} e^{\mu} \\ &= ne^{\mu}. && (10) \end{aligned}$$

Thus 1 becomes

$$D = \log\left(\frac{1}{b}\mu + \frac{a}{b} - ne^{\mu}\right). \qquad (11)$$

Differentiating with respect to μ yields

$$\frac{dD}{d\mu} = \frac{\frac{1}{b} - ne^{\mu}}{\frac{\mu}{b} + \frac{a}{b} - ne^{\mu}} \tag{12}$$

$$\approx 1, \tag{13}$$

since the e^{μ} terms dominate all others in 12 for sufficiently large values of μ.

References

[1] Fenton L. The sum of log-normal probability distributions in scatter transmission systems. IRE Transactions on communications systems. 1960;8(1):57–67. doi:https://doi.org/10.1109/tcom.1960.1097606.

[2] Cobb BR, Rumi R, Salmerón A. Approximating the distribution of a sum of log-normal random variables. Statistics and Computing. 2012;16(3):293–308.
