# Supplementary material for: Difficulties in summing log-normal distributions for abundance and potential solutions
Source: PLoS One. 2023 Jan 12;18(1):e0280351. doi: 10.1371/journal.pone.0280351 (PMC9836268; doi:10.1371/journal.pone.0280351)
Supplement: S2 Text — (PDF) [file pone.0280351.s005.pdf]

S5 Text for

Difficulties in summing distributions for abundance and potential solutions

Emma Talis[1,2], Christian Che-Castaldo[2], Heather J. Lynch[2,3]

[1]Department of Applied Mathematics and Statistics, Stony Brook University

[2]Institute for Advanced Computational Science, Stony Brook University

[3]Department of Ecology and Evolution, Stony Brook University

How Bayesians interpret the tail of the skewed distributions

For simplicity, we limited our manuscript's discussion on the mechanics of summing distributions for population abundance. However, it is also worth reflecting on our interpretation of the posterior distribution and, specifically, on the interpretation of the log-normal distribution's long right tail. One interpretation of this long right tail is that such abundances are infrequent and thus including them in the aggregate sum (at the appropriately small probability) is appropriate. A more Bayesian interpretation would be that such tail events are not infrequent but are instead *unlikely to be true* (in the "degree-of-belief" sense), in which case they should *not* be included in the aggregate total. While extreme outcomes (i.e. those in the right tail of the distribution) are by nature rare, the probability of drawing at least one extremely large abundance when summing across posteriors can be quite high, and eventually inevitable (as illustrated in Fig 1C in the main text), meaning the aggregate abundance will almost certainly include at least one very large draw, and thus will be larger than anticipated by the central tendencies of the individual populations. The interpretation of the Bayesian posterior in this context deserves discussion but falls outside the scope of our manuscript, which was written for an audience of conservation practitioners.
