# Supplementary material for: Difficulties in summing log-normal distributions for abundance and potential solutions
Source: PLoS One. 2023 Jan 12;18(1):e0280351. doi: 10.1371/journal.pone.0280351 (PMC9836268; doi:10.1371/journal.pone.0280351)
Supplement: S3 Text — (PDF) [file pone.0280351.s006.pdf]

S6 Text for

Difficulties in summing distributions for abundance and potential solutions

Emma Talis[1,2], Christian Che-Castaldo[2], Heather J. Lynch[2,3]

[1]Department of Applied Mathematics and Statistics, Stony Brook University

[2]Institute for Advanced Computational Science, Stony Brook University

[3]Department of Ecology and Evolution, Stony Brook University

Summing negative binomial distributions

Consider a collection of $n = \{10, 100, 1000\}$ independent and identically negative binomially-distributed populations of animals, each with an abundance that is modeled as $N_{i,t} \sim NB(\mu, k)$ where μ is the mean of abundance, varying between 2,000 and 160,000, and k is the overdispersion ("size") parameter, fixed at 4. (The overdispersion parameter measures the amount of clustering, or aggregation, or heterogeneity in the data: a smaller k means more heterogeneity; when $k = 0$, the NB distribution is equivalent to the Possion distribution.) Each population consists of $m = 1000$ negative binomially-distributed draws. If an estimate for the total abundance across all of these populations (the "regional" abundance) is sought, then the distribution of interest, that of the regional abundance, is thus the sum of many log-normal distributions. We first consider the choice of summary statistic for a single negative binomial distribution, and then consider the differences between methods of summing across multiple distributions. Similar to that of the log-normal distribution, the mean of a negative binomial random variable is pulled toward the extreme values of the distribution's long right tail and, as a result, the mean is always larger than the median (Fig 1).

We now consider summing across multiple populations whose abundances are described each by a negative binomial distribution. Unlike the case of the log-normal distribution, the probability

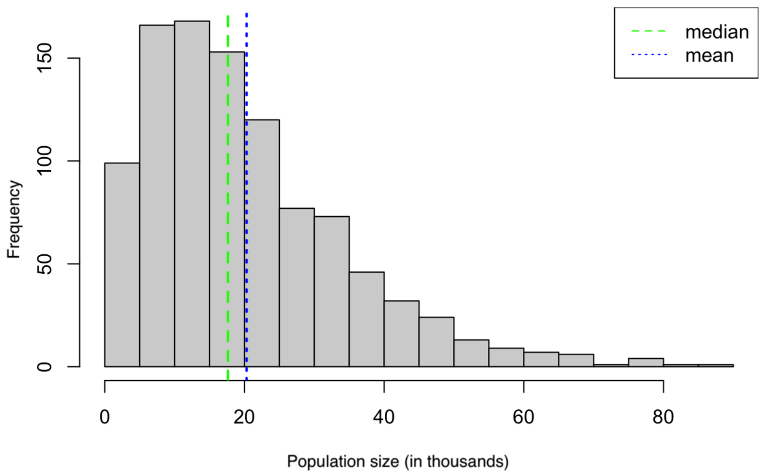

Figure 1: Negative binomial distribution for abundance with $\mu = 20,000$ and $k = 2$.

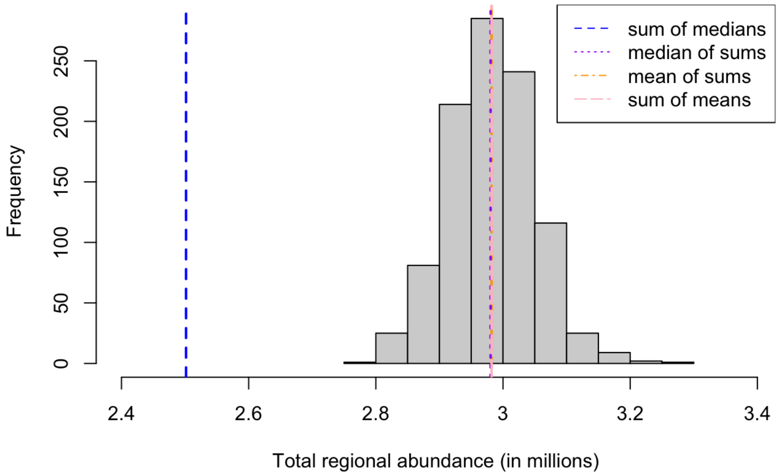

Figure 2: The distribution of $m = 1000$ sums of NB-distributed abundance samples across $n = 1000$ independent populations. Each NB-distributed population has mean abundance $\mu = 4000$ and overdispersion parameter $k = 2$. The values of the sum of the medians, median of sums, and mean of sums (which is equal to the sum of the means) are shown.

generating function of the sum of n negative binomial random variables is known in closed form.

The sum $S = X_1 + X_2 + ... + X_n$ where each X_i follows the negative binomial distribution has been shown to be a mixture binomial random variable [1, 2]. However, we show in Fig 2 that sums of negative binomial random variables behave similarly to those of log-normal random variables when

the median is used as the measure of central tendency. If the median is used as a point estimate of abundance, it follows that either the median of the sums or the sum of the medians represents the best estimate of the aggregate abundance. However, since the median of sums is not the sum of medians, these two methods give different estimates for total abundance across the region, with the latter approach (sum-then-summarize, giving the median of the sums) yielding a significantly larger estimate of total abundance than former (summarize-then-sum, giving the sum of the medians), as shown in Fig 3. This phenomenon is similar to that described for the log-normal distribution.

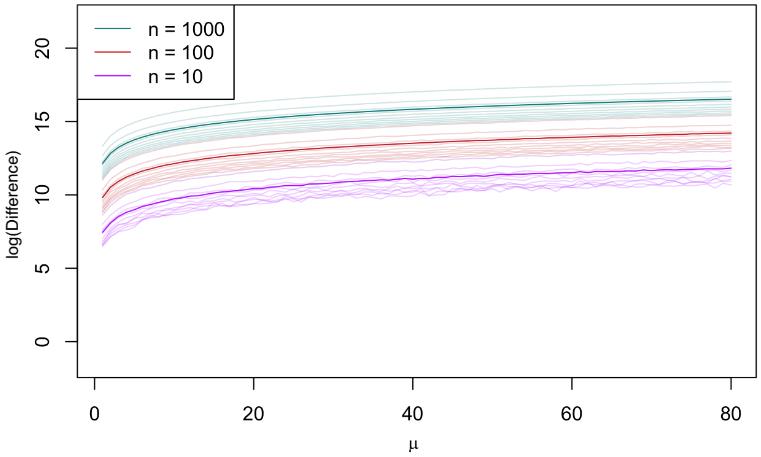

Figure 3: The logged difference in global abundance estimates for $n = \{10, 100, 1000\}$ i.i.d. negative binomial-distributed populations is plotted against the mean abundance μ. Solid lines represent the mean of each set of 10 ensembles. For each simulation, we draw $m = 1000$ samples for each population and calculate the difference between the median of the sample-wise aggregated regional population and the sum of the empirical population medians.

References

[1] Furman E. On the convolution of the negative binomial random variables. Statistics & probability letters. 2007;77(2):169–172. doi:https://doi.org/10.1016/j.spl.2006.06.007.

[2] Chen X, Guisong C. Exact distribution of the convolution of negative binomial random variables. Communications in Statistics-Theory and Methods. 2017;46(6):2851–2856. doi:https://doi.org/10.1080/03610926.2015.1053931.
